# Hectogram-Scale Synthesis of Carbamates Using Electrochemical Hofmann Rearrangement in Flow

**DOI:** 10.1021/acs.oprd.5c00234

**Published:** 2025-08-12

**Authors:** Darryl F. Nater, Rong Zhao, Johannes Rocker, Coline Boche, Dabeen Yun, Bernd Werner, Patrick Löb, Athanassios Ziogas, Siegfried R. Waldvogel

**Affiliations:** † 28313 Max-Planck-Institute for Chemical Energy Conversion, Department of Electrosynthesis, Stiftstraße 34−36, Mülheim an der Ruhr 45470, Germany; ‡ 33437 Boehringer Ingelheim Pharma GmbH & Co. KG, Binger Straße 173, Ingelheim am Rhein 55218, Germany; § 42227 Fraunhofer Institute for Microengineering and Microsystems IMM, Carl-Zeiss-Straße 18-20, Mainz 55129, Germany; ∥ Karlsruhe Institute of Technology (KIT), Institute of Biological and Chemical Systems - Functional Molecular Systems (IBCS-FMS), Kaiserstraße 12, Karlsruhe 76131, Germany

**Keywords:** electrosynthesis, bromide mediation, scale-up, Hofmann degradation, sandwich cell, glassy carbon

## Abstract

The Hofmann rearrangement of alkyl and aryl carboxamides can be achieved electrochemically on a hectogram scale. The necessary halogen species and base equivalents are generated from sodium bromide in methanol by electrolysis. Both the supporting electrolyte and the solvent fulfill the role of mediator, conductivity-enabling agent, and reaction partners. This electrochemical conversion can be carried out in simple and commercially available plate-frame cells if a simple glassy carbon anode is used. A productivity of 104 mmol per h is obtained with 162 cm^2^ of anode surface.

## Graphical abstract

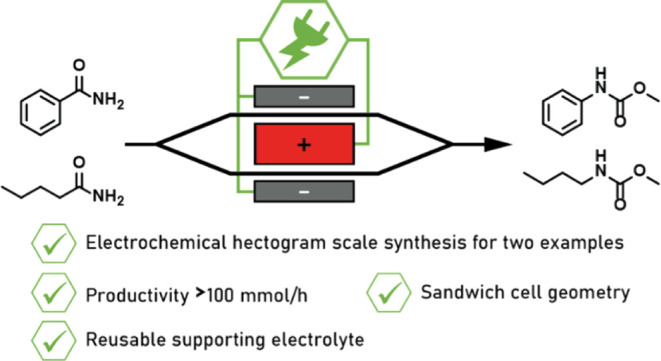

## Introduction

The sustainability of chemical processes has become a major focus for both academia and industry over the past few decades. While carbon emissions are often foremost in discussions of sustainability, other factors are just as important, as illustrated, for example, by the 12 principles of green chemistry.^1^ Among the goals set by these principles is the avoidance of hazardous compounds in synthesis. However, many critical processes and their products rely on such reactive reagents, making the development of alternative synthesis procedures a major focus of both industrial and academic laboratories.^2^ A prominent example of the use of hazardous substances in synthesis is the generation of carbamates from amides by Hofmann rearrangement.^3^ This reaction requires (super-) stoichiometric amounts of caustic halogen or halo-reagents.^4–9^

The generated carbamates are very versatile and can either be converted into amines or directly find diverse applications throughout the chemical industry. They are widely used in pharmaceutical chemistry,^10–14^ wherein they are linked to increased biological activity in active ingredients. Additionally, they are used in agrochemical^15,16^ and fine chemical applications,^17^ since their versatility as linkers is highly useful toward derivatization efforts.^18,19^ Seeing the critical importance of this transformation, finding an alternative approach to this process is of major interest. Lowering environmental impact is highly desired.

Electrosynthesis has garnered increased interest in the past few years as a methodology that allows for the replacement of hazardous reagents by generating them in situ.^20–29^ The anodic generation of reactive halogen species represents an interesting way to initiate reactions or generate strong oxidizers.^30–33^ This approach has also been applied to rearrangement reactions. In the case of the Hofmann rearrangement, the required halo reagent is formed in situ by the oxidation of halide, whereas the other necessary reactant, a base, is generated cathodically by concomitant hydrogen production.^34^ The resulting process features a halo source that is safe to handle, atom-efficient, and can even be recovered from the reaction mixture, allowing for direct reuse and further minimizing the waste streams generated by such processes. Noteworthily, the bromide salt serves a dual role as both mediator and supporting electrolyte.^35^ This reaction has been conducted in various cell types including simple batch-type cells,^34^ parallel plate reactors,^36^ and spinning anode electrolyzers.^37^ (Figure 1) The hydrogen evolution and concomitant base formation serve as the counter reaction. The counter reaction at the cathode interacts strongly within the transformation, as it provides the base necessary to initiate the desired reaction pathway.^38^

**Figure 1:**
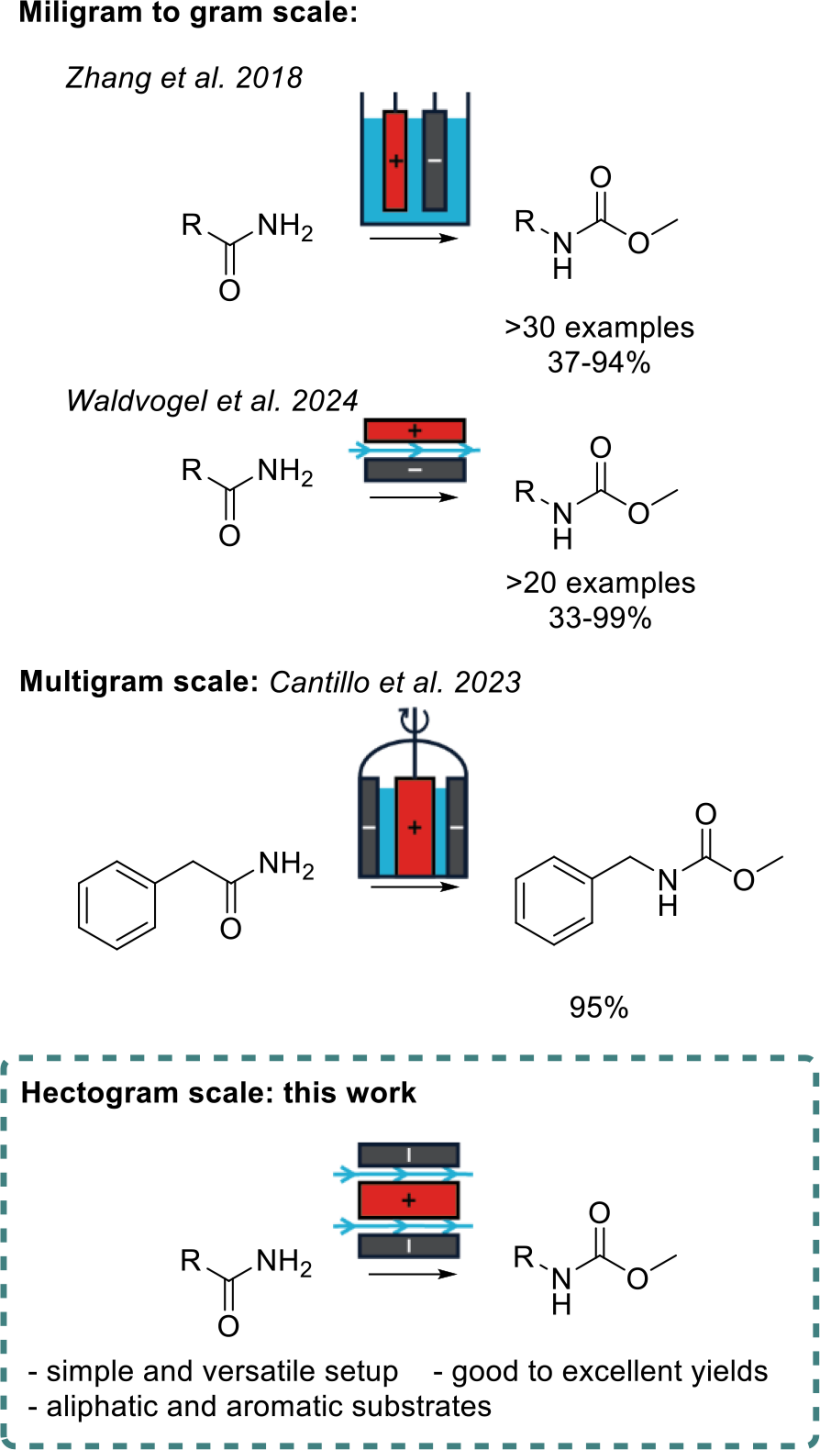
Different cell types and scales employed in the electrochemical Hofmann rearrangement.

While previous studies have been conducted on the electrochemical Hofmann rearrangement, they have focused on batch sizes ranging from milligrams to a few grams. However, due to the plethora of possible applications for carbamates, their synthesis also needs to be scrutinized at larger scales. These investigations are of particular importance for novel synthesis technologies such as electrosynthesis. Additionally, this is especially important as this transformation utilizes both cathodically and anodically generated species. As such, it is highly susceptible to the geometry of the cell.

Accordingly, we were interested to see whether we could increase the scale of this process to the hectogram scale while maintaining the excellent yields that have been observed on smaller scales.^36^ As such, our further development of this electrosynthetic method aims to significantly increase the accessibility and applicability of electrosynthetic reactions at scale. In addition, the use of a commercially available cell and parts facilitates the adoption of this technique by other laboratories as well.^39,40^

## Results and Discussion

Due to their simplicity and versatility, we decided to continue using parallel plate reactors^41^ for our investigation, and in order to keep the resulting process accessible, we used a commercially available cell with stainless steel as the cathode and glassy carbon as the anode.

This reactor has two separate but undivided compartments, each of which has an available anode surface area of 81 cm^2^. Due to it is a sandwich cell design with the anode as the center, only one glassy carbon electrode is necessary to reach a total anode surface of 162 cm^2^. (Figure 2) In order to validate the electrochemical Hofmann rearrangement, we performed reactions in one compartment of the cell using the same current density and residence time as previously reported with smaller cells^36^ while increasing the scale to 20 mmol of starting material. Performing this reaction with benzamide (**1**) resulted in an isolated yield of 62% of the corresponding methyl carbamate (**2**), or approximately 2/3 of the yield reported for a parallel plate reactor with 12 cm^2^ of anode surface under comparable conditions. Since a higher yield should be possible in this larger cell, we optimized the reaction for benzamide toward maximum yields by changing current density, sodium bromide concentration, interelectrode distance, and flow rate.

**Figure 2:**
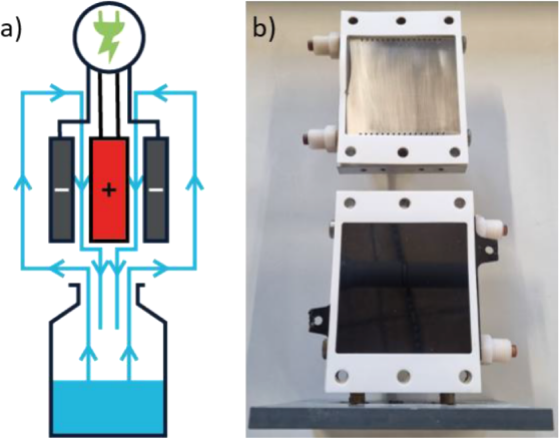
(a) Schematic display of the electrolysis setup composed of an electrochemical sandwich flow cell, with the electrolysis compartments working in parallel. (b) Picture of the cell with one electrolysis compartment opened. Top: stainless-steel cathode; bottom: glassy carbon anode.

This investigation indicated that, in order to maximize yields, we need to decrease the current density and the interelectrode gap while increasing the bromide concentration. The flow rate was found to have only a negligible influence. Under these conditions, we could still observe residual starting material and thus investigated using higher amounts of applied charge, with full conversion being achieved when 4 *F* were employed. Additionally, we found that the reaction mixture needed to be stirred for at least 2 h after completion of the electrolysis to achieve the maximum yield (see Supporting Information).

By adjusting our process accordingly, we achieved a maximum yield of 88% for the conversion of benzamide (**1**) to methyl *N*-phenylcarbamate (**2**) (Scheme 1). With an established method for an aromatic starting material, we subsequently investigated valeramide (**3**) as a representative aliphatic substrate. Using the previously determined optimal conditions as a starting point, we investigated the influences of current density, interelectrode distance, and bromide concentration on the yield of methyl *N*-butylcarbamate (**4**). As the flow rate had only negligible influence in our preceding optimization, we removed this factor from the study. Our investigation showed a tendency toward higher yield with increased current density and interelectrode distance, culminating in an almost quantitative yield of 98% for **4** (Scheme 2).

**Scheme 1:**
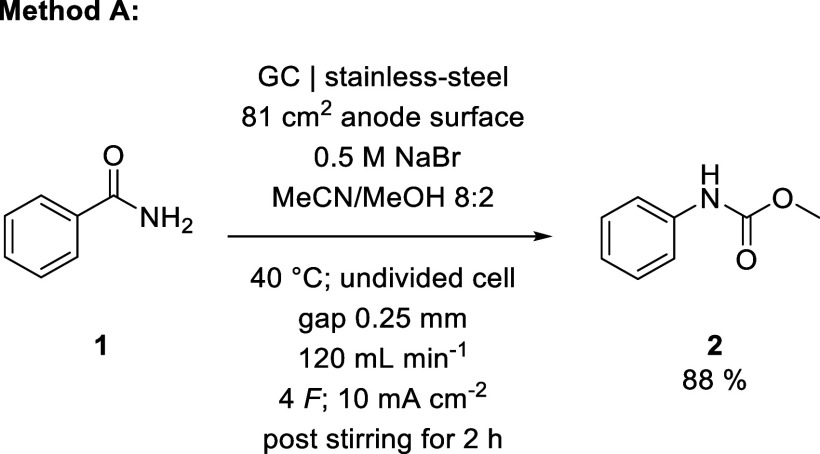
Electrolysis Conditions Optimized for Yield of Methyl *N*-Phenylcarbamate

**Scheme 2:**
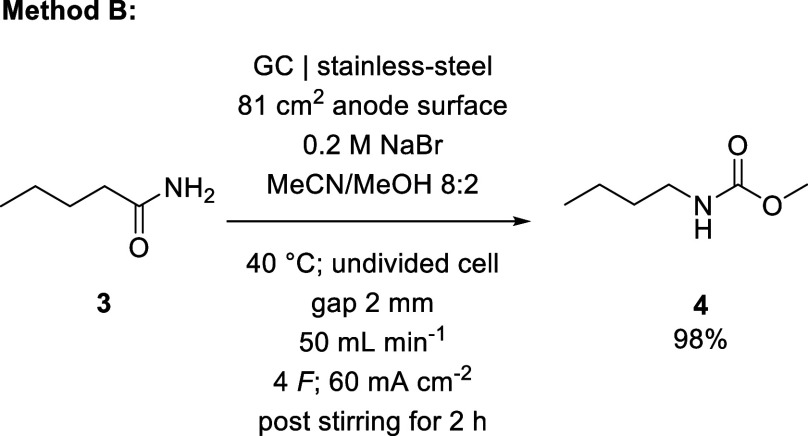
Electrolysis Conditions Optimized for Yield of Methyl *N*-Butylcarbamate

Interestingly, the influences of current density, bromide concentration, and interelectrode gap all showed inverted trends between the investigated aromatic and aliphatic substrates. However, both substrates required post-stirring for at least 2 h after the end of the electrolysis in order to complete the transformation. This indicates that the electrochemical generation of a bromo reagent is faster than the subsequent chemical steps. No intermediates were observed, and the rate-determining step may either be the deprotonation and bromination of the starting material or the subsequent deprotonation leading to the rearrangement. The isocyanate, once formed, is believed to rapidly produce the carbamate based on literature-reported reaction constants.^42^

We subsequently compared the conditions employed in the smaller flow cell with 12 cm^2^ anode surface^36^ and our larger system with 81 cm^2^ anode surface. While the yields of **2** are comparable, the current density and interelectrode distance needed to be drastically reduced, and the amount of bromide mediator increased for the larger cell. The combination of these factors led to an increase in productivity to 26 mmol/h of product, which corresponds to a 3.1-fold increase compared to the previously used parallel plate reactor. Simultaneously, in the case of valeramide, the current density could be slightly increased, which, coupled with a slight decrease in the amount of applied charge and an increased yield in the larger reactor, resulted in a productivity of 46 mmol/h for **4**, corresponding to a more than 10-fold increase compared to the previously employed setup. Additionally, this productivity could be doubled by using both sides of the sandwich cell. The yield-oriented optimization thus led to a method with high productivity for the aliphatic product, which is desirable for the synthesis of the carbamate at scale. Meanwhile, the aromatic substrate could only be produced in optimized yields by sacrificing productivity, making the method less attractive for large-scale carbamate synthesis.

As such, we needed to reinvestigate our method for the synthesis of aromatic carbamates with a focus on productivity. Accordingly, we investigated the rearrangement of benzamide with a focus on the current density, interelectrode distance, flow rate, and starting material concentration. During this optimization step, all reactions were supplied with charge until full conversion of the starting material was observed by TLC. To reach high productivity, we found both high current density and low interelectrode distances to be of paramount importance, with smaller improvements enabled by high flow rates and low starting material concentrations (Scheme 3). However, as previously observed, higher current densities led to lower yields; as such, we needed to find a current density that would result in high productivity without losing too much in yield. Following this design principle, we landed on conditions C, which give the product in 81% yield, corresponding to a productivity of 52 mmol/h of **2**, or twice that of the yield-optimized procedure (Scheme 1).

**Scheme 3:**
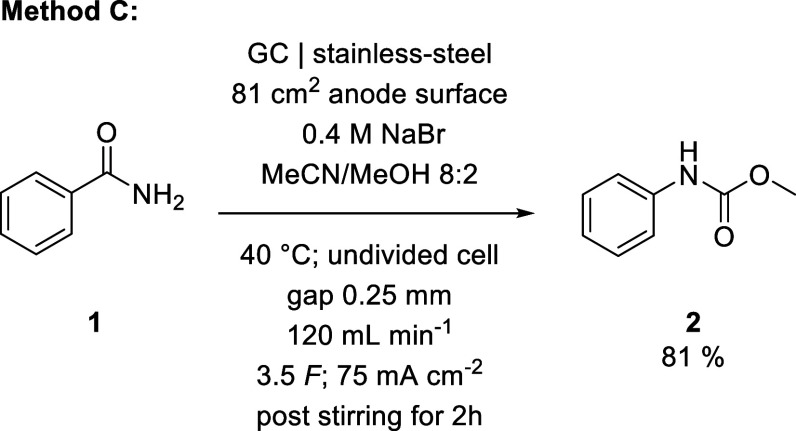
Reaction Conditions Optimized for Productivity of Methyl *N*-Phenylcarbamate

With a focus on productivity and utility, we further investigated method B for the transformation of **3** (Table 1). While it would be desirable to perform the reaction at higher substrate concentrations, we found that doubling the concentration of starting material led to a decrease in yield to 85%.

**Table 1:** Investigations to Further Increase the Productivity and Practicability of Method B

Deviation from method B	Yield
None	98%
Double substrate concentration	85%
90 mA/cm^2^ current density	90%
120 mA/cm^2^ current density	88%
Reservoir at rt	97%

Methods B and C show good to excellent yields and productivities; as such, we investigated both of them using both reaction compartments of the cell. As the yields did not change significantly for either method, we investigated the reaction mixtures after electrolysis more in depth.

Similarly, even higher current densities of 90 and 120 mA/cm^2^ also led to decreased yields of 90% and 88%, respectively. Finally, the reaction was performed at room temperature, which resulted in a yield of 97%. As such, performing the reaction at higher current densities and higher concentrations will result in slightly decreased yields. However, the losses are manageable, making the consideration of running the reactions at higher current densities or concentrations worthwhile.

When investigating the electrolysis mixtures obtained using methods A and C, we found the desired products in the previously mentioned yields. However, we also found methyl phenyl ester as a byproduct in 5% yield in both cases. Furthermore, bromo-substituted phenyl carbamates could also be identified in 5% yield for method A and 13% for method C. This difference implies that, at higher current densities and concomitantly higher productivities, bromination becomes a more prevalent side reaction. The remaining 1% and 2% of starting material could not be attributed to a species in the final reaction mixture. When investigating the electrolysis mixture from method B, we only observed methyl butyl ester as a byproduct, with no indication of bromination as a byproduct. (Figure 3)

**Figure 3:**
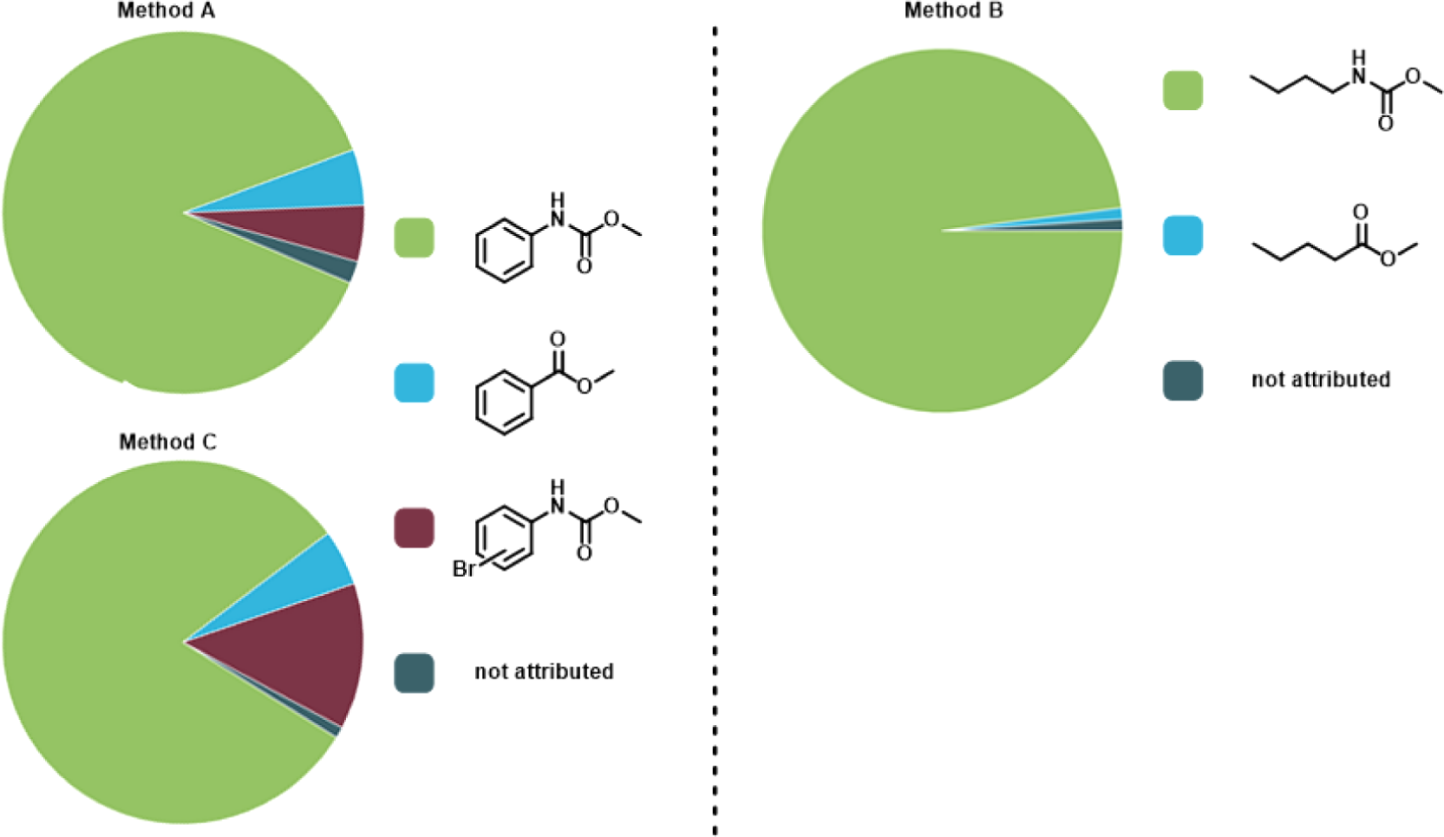
Carbon balance of products and byproducts from electrolyses using methods A, B, and C.

To fully illustrate the applicability of our developed methods, we aimed to perform synthesis on a hectogram scale for both substrates.

The reaction of **1** was performed using method C with 1 mol of starting material and required an electrolysis time of 8 h (Scheme 4).

**Scheme 4:**
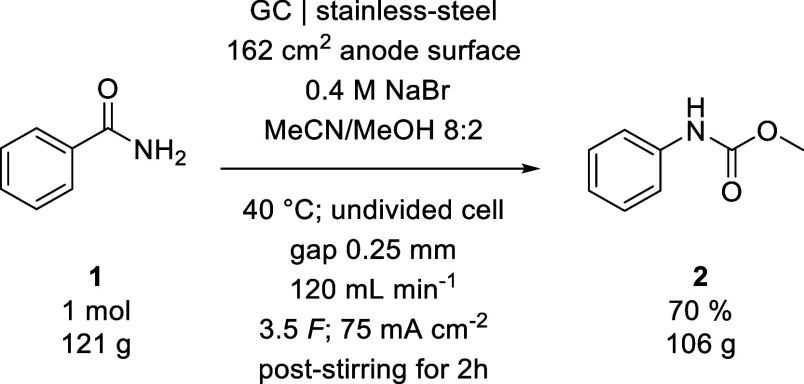
Reaction Conditions Used for the Hectogram Synthesis of **2**

While the electrolysis was ongoing, we monitored both the cell temperature and the product yield (Figure 4). The cell temperature was stable around 37 °C during the entire electrolysis, which is slightly below the temperature of the reservoir at 40 °C. The yield initially increased nearly linearly for the first 3 h, after which a decrease in the generation of **2** could be observed. This behavior could be due to the bromination reaction becoming more prominent at higher product concentrations. After the electrolysis and stirring overnight, a total of 106 g of **2** could be isolated by crystallization, representing a final yield of 70%.

**Figure 4:**
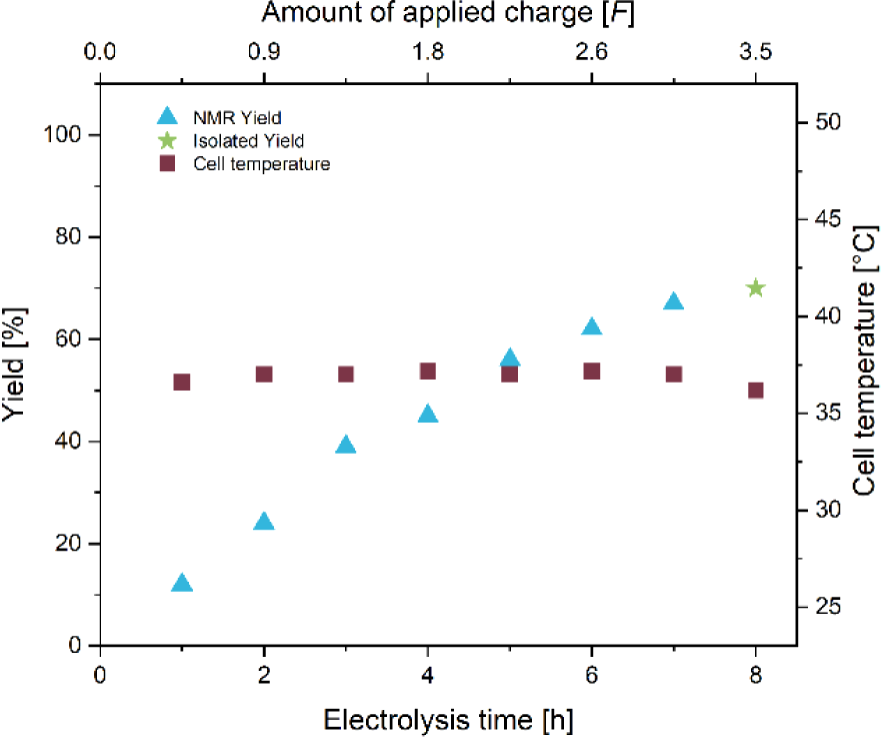
Cell temperature and yield during the hectogram synthesis of **2**.

The reaction of **3** was also performed on a hectogram scale using method B with 1 mol of starting material and the reaction reservoirs at room temperature, which required a total electrolysis time of 10.7 h. (Scheme 5)

**Scheme 5:**
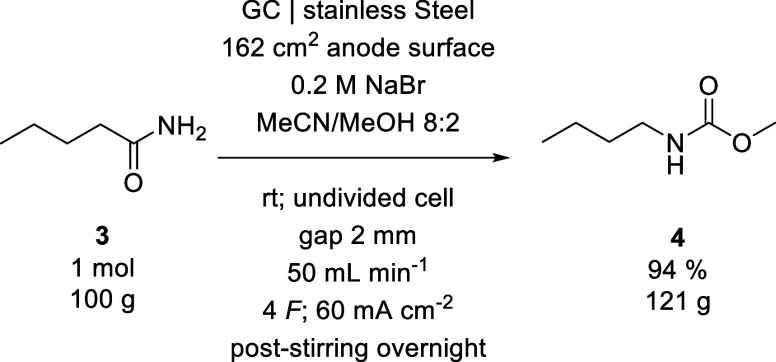
Reaction Conditions Used for the Hectogram Synthesis of **4**

During the reaction, both the cell temperature and the yield were monitored hourly (Figure 5). After an initial period of temperature increase, the cell temperature stabilized at just above 33 °C. The monitoring of the yield showed a nearly linear increase in the concentration of **4**, with a final isolated yield of 94% at the end of the electrolysis.

**Figure 5:**
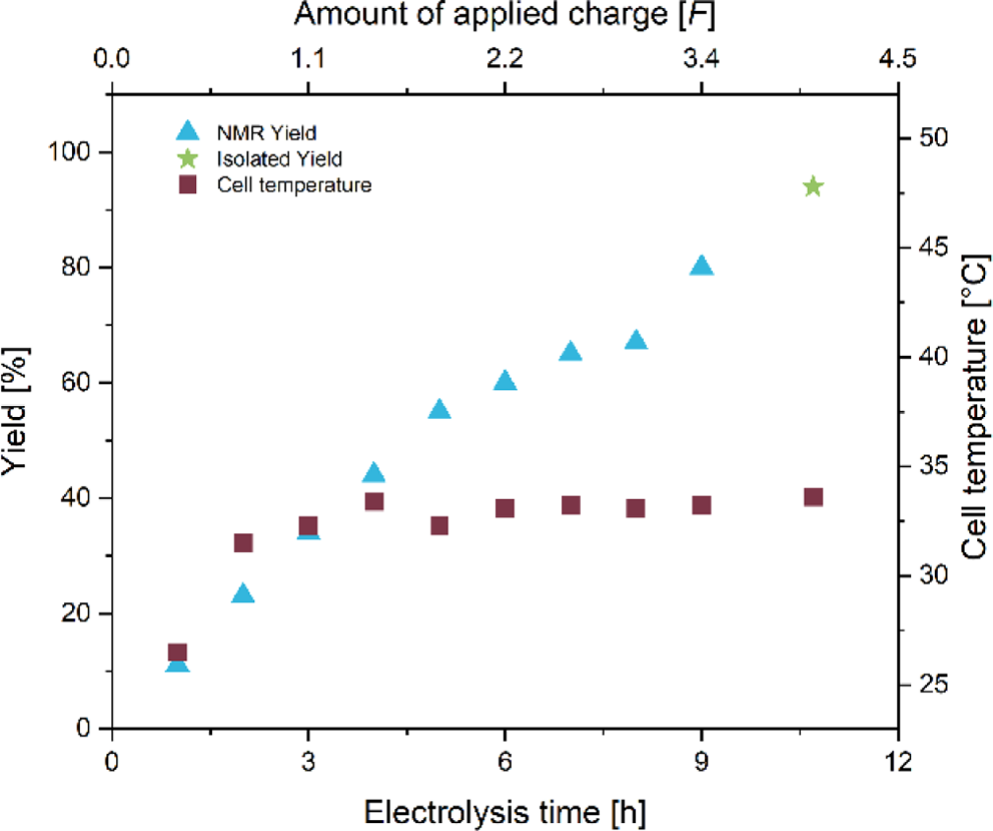
Cell temperature and yield during the hectogram synthesis of **4**.

While this yield is slightly lower than the one observed during the optimization, investigation of the residue after the distillation of the product still showed residual starting material, indicating that near-quantitative yields might be reached by slightly increasing the amount of applied charge.

Both of these methods have shown applicability even on large scales, and the employed bromide salt could be recovered at >95%. However, for the aromatic substrate, we still observed significant amounts of byproducts, which might originate from local fluctuations in temperature or an uneven distribution of flow in the cell. As such, we designed an alternative parallel plate reactor with a sandwich architecture and 100 cm^2^ of an anode surface per side. The electrode materials remained the same, with a glassy carbon plate serving as the anode for both reaction compartments and cathode plates made from stainless steel. Initial models showed that the flow throughout the cell would be uneven at this size (Figure 6a), which prompted us to adjust the inlet channels to provide an even solvent velocity across the entire cell (Figure 6b; details see Supporting Information)

**Figure 6:**
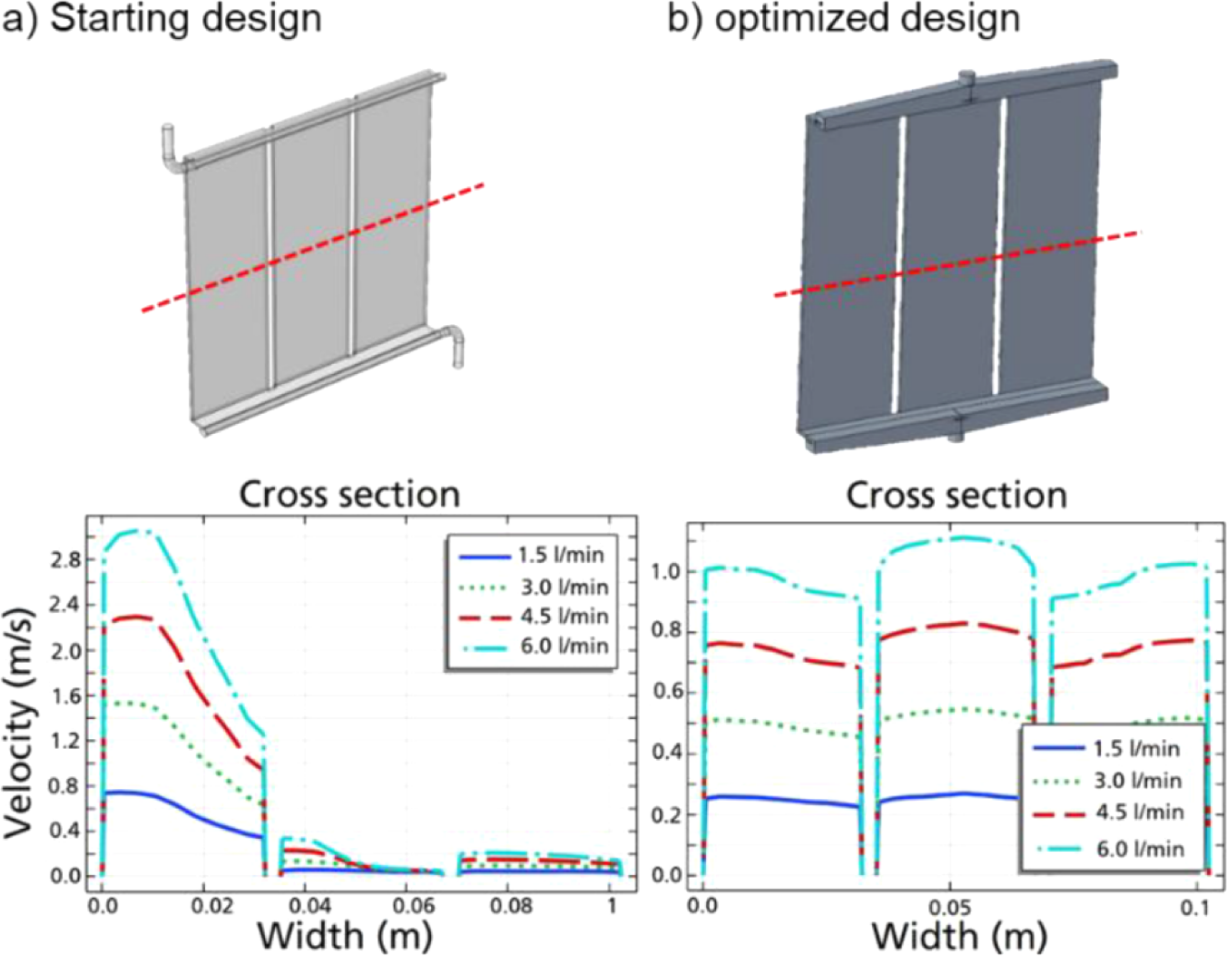
Flow fields of the starting (a) and optimized (b) design of the custom-built parallel plate reactor, and modeled profiles of liquid velocity in (a) and (b) at cross sections at the red dotted lines.

Additionally, to better control the cell temperature, the cathode plates were equipped with a heat exchanger. After some preliminary experiments (see Supporting Information), we revisited the reaction of **1** using this cell and investigated the rearrangement on the 500 mmol (61 g) scale, resulting in method D (Scheme 6 and Table 2).

**Table 2:** Investigations to Improve Method D at Higher Reaction Scales

Deviation from method D	Yield **2** (%)	Brominated products (%)
none	94	3
100 mA/cm^2^, 4 *F*	85	12
100 mA/cm^2^, 10 *F*	68	22
2.4 L/min, 4.5 *F*	93	4
1200 mmol, 7 *F*	82	0

**Scheme 6:**
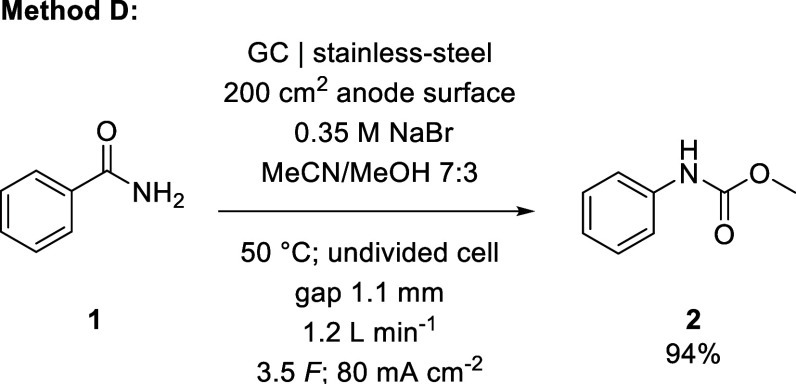
Reaction Conditions Optimized for Yield of Methyl *N*-Phenylcarbamate in the Custom-Built Reactor

During these investigations, we observed a marked decrease in yield when the current density was further increased to 100 mA/cm^2^, which simultaneously required an increase in applied charge to achieve full conversion. However, even larger increases in applied charge, up to 10 *F*, resulted in both lowered yield and more brominated byproducts. This indicates that the rearrangement is strongly favored at the beginning of the electrolysis, but the side reaction becomes more prevalent as the reaction proceeds and continues to consume the product when the starting material is depleted. Close monitoring of the reaction progress is, therefore, essential to avoid side product formation and product degradation, particularly with sensitive substrates.

Further investigations into the flow rate showed that it had only a minor influence on yield, as doubling the flow rate did not significantly change the yield. However, the reaction at a lower flow rate was found to have slightly improved reaction kinetics, as it reached full conversion after 3.5 *F* of applied charge compared to the 4.5 *F* necessary at 2.4 L/min. Interestingly, at these high flow rates, no stirring after completion of the electrolysis was necessary to reach the maximum yields, which is attributed to better mixing in the electrolyte vessel and the higher reaction temperature during electrolysis.

The conversion of **3** was also investigated in this new reactor, with the optimized method (Scheme 7) providing 98% yield of the corresponding carbamate **4** with a marked decrease in the formation of the ester byproduct, which was attributed to the increased cell temperature (Figure 7).

**Scheme 7:**
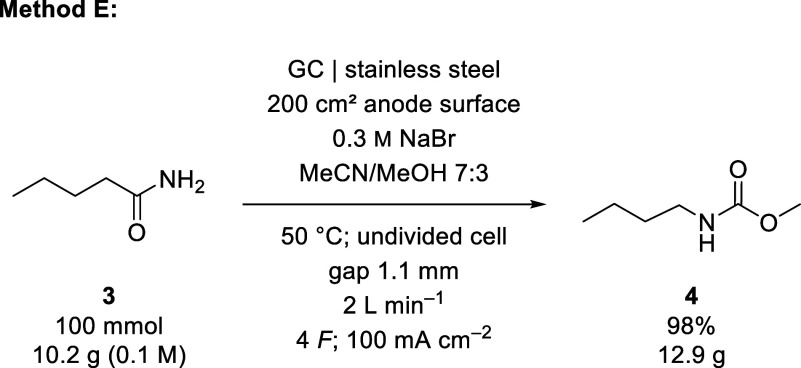
Reaction Conditions Optimized for Yield of Methyl *N*-Butylcarbamate in the Custom-Built Reactor

**Figure 7:**
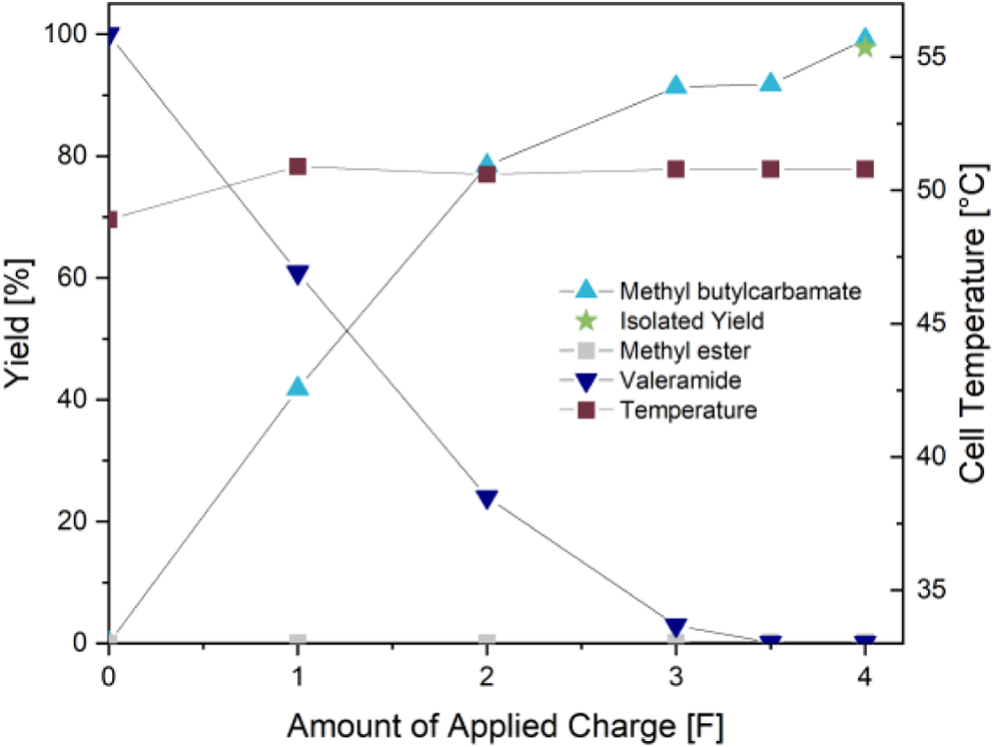
Cell temperature and yield during the synthesis of **4** on a 10 g/100 mmol scale.

Compared to method B, we could also significantly increase the current density to 100 mA/cm^2^ and accordingly the productivity to 182 mmol/h.

## Conclusion

In conclusion, we have shown that the electrochemical Hofmann rearrangement can be performed on a hectogram scale using both a commercial electrolysis cell and one specifically designed for the process. Both cells allowed for good to excellent yields and productivities. Substrates that are liable to bromination were found to be more problematic, as the formation of brominated byproducts competes with the desired rearrangement.

This study underscores the possible uses of electrosynthesis, even on larger scales. Simultaneously, we show that changes to an electrochemical synthesis method also need to be tailored to the substrate, not just the reaction. However, these adjustments are possible by using modular electrochemical cells and motivate the use of versatile electrochemical setups.

## Supplementary Material



## Notes

Detailed descriptions of the employed setup and the experimental conditions are available in the Supporting Information (PDF)
