# Supplementary material for: Hectogram-Scale Synthesis of Carbamates Using Electrochemical Hofmann Rearrangement in Flow
Source: Org Process Res Dev. 2025 Aug 12;29(9):2370–7. doi: 10.1021/acs.oprd.5c00234 (PMC12455659; doi:10.1021/acs.oprd.5c00234)
Supplement: Supplementary file 1 [file op5c00234_si_001.pdf]

Hectogram Synthesis of Carbamates using Electrochemical Hofmann Rearrangement in Flow

Darryl F. Nater,[1] Rong Zhao,[2] Johannes Rocker,[3] Coline Boche,[1] Dabeen Yun,[1] Bernd Werner,[2] Patrick Löb,[3] Athanassios Ziogas,[3] and Siegfried R. Waldvogel[1,4]*

[1]Max-Planck-Institute for Chemical Energy Conversion, Stiftstraße 34–36, 45470 Mülheim an der Ruhr (Germany)

[2]Boehringer Ingelheim Pharma GmbH & Co. KG, Binger Straße 173, 55218 Ingelheim am Rhein (Germany)

[3]Fraunhofer Institute for Microengineering and Microsystems IMM, Carl-Zeiss-Straße 18-20, 55129 Mainz (Germany)

[4]Karlsruhe Institute of Technology (KIT), Institute of Biological and Chemical Systems - Functional Molecular Systems (IBCS-FMS), Kaiserstraße 12, 76131 Karlsruhe (Germany)

e-mail: siegfried.waldvogel@cec.mpg.de

---

1. General Information S2
2. Electrochemical Cells S2
2.1 Commercial Cell S2
2.2 Custom-built Cell S4
3. Optimization of Reaction Conditions S5
3.1 General Reaction Protocol for Optimization S5
3.2 Initial Reaction S5
3.3 Influence of Post-stirring Time on Yield S5
3.4 Method A S6
3.5 Method B S7
3.6 Method C S8
3.7 Method D S10
3.8 Method E S11
4. Synthesis of Carbamates at Hectogram Scale S11
4.1 Hectogram Synthesis of Methyl *N*-phenylcarbamate S11
4.2 Hectogram Synthesis of Methyl *N*-butylcarbamate S12
5. Full Comparison of Different Flow Reactors used for the Electrochemical Hofmann Rearrangement S13
6. NMR Spectra of Products S13
7. References S18

1. General Information

All materials were obtained, unless otherwise noted, from commercial suppliers and not further purified.

^{1}H and ^{13}C NMR spectra were recorded by a Bruker Avance II 400 (440 MHz) or a Magritek Spinsolve Multi-X 80MHz. Chemical shifts were reported in parts per million (ppm) relative to tetramethyl silane (TMS) at 0 ppm or residual $HCCl_3$ at 7.26 ppm.

Gas chromatography was performed using an Agilent 7890B GC with a DB-35MS column. Quantification of the FID signals was performed by calibration with 4-methoxybenzophenone as internal standard.

Either a HMP4040 (Rhode&Schwartz) programmable power supply or a P1570 (PeakTech) programmable power supply was used as a DC powersource. All electrolysis was executed under galvanostatic conditions.

Fluid simulations were performed in COMSOL 6.3 assuming methanol as fluid and an electrical current of 10 A at the cathode resulting in hydrogen evolution during passage.

2. Electrochemical Cells

2.1 Commercial Cell

The commercial electrochemical cell used was a Condias SZ100 Sandwich cell[1] with a glassy carbon plate (Sigradur® G, HTW Hochtemperatur-Werkstoffe GmbH) as the central anode. Each anode side had an active surface area of 81 cm^2.

When assembling the cell, one of the cathodes was equipped with six screws (Figure S1a). Subsequently, Teflon spacers (ranging in thickness from 0.25 mm to 1 mm) were threaded onto the screws on top of the stainless-steel cathode, leaving an 81 cm^2 area exposed (Figure S1b).

Following this, two peek bars were added on top of the spacers (Figure S1c) and the anode placed between them (Figure S1d). Afterwards, another set of Teflon spacers was added (Figure S1e) followed by the installation of the second cathode, which was secured with a set of nuts (Figure S1f).

Lastly, the inlet and outlet and outlet of the cell were connected to 10 mm OD PTFE tubing using a Teflon joint.

A graphical summary of the parts used in the cell can be found in Figure S1g.

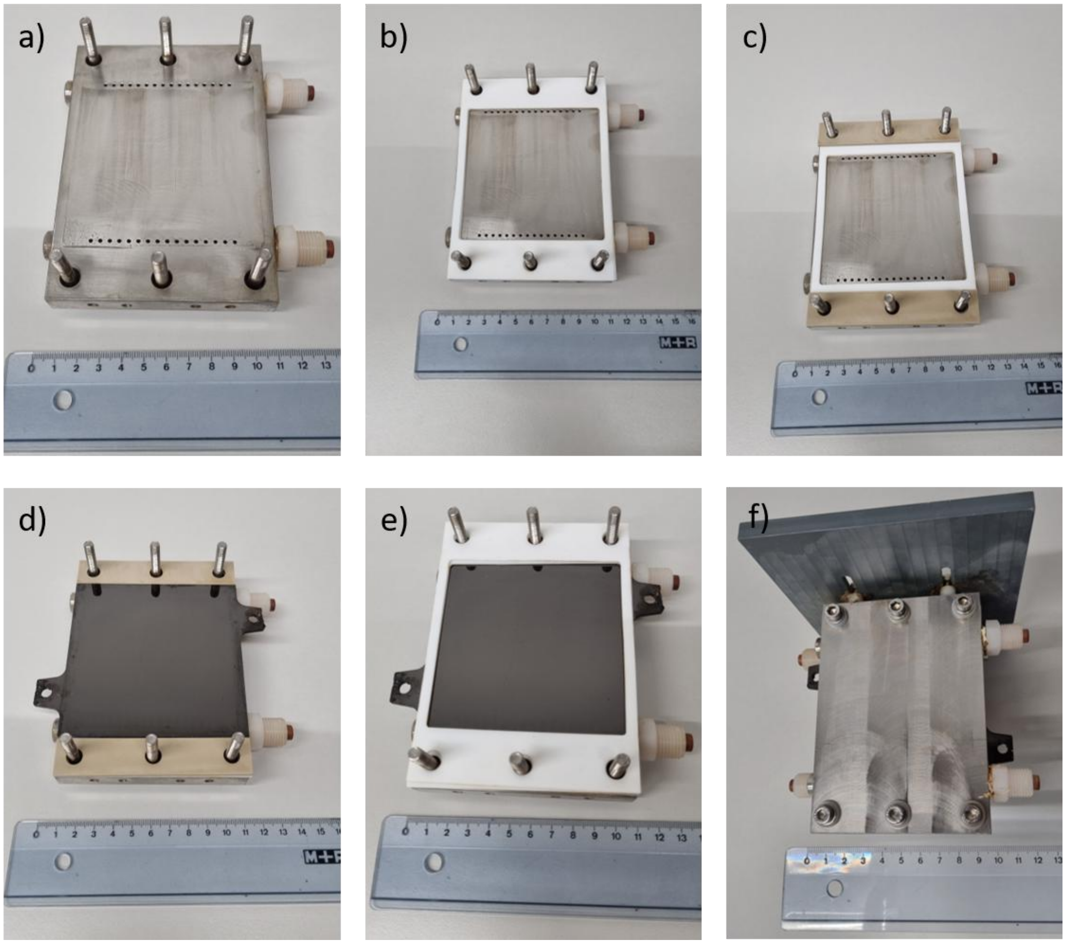

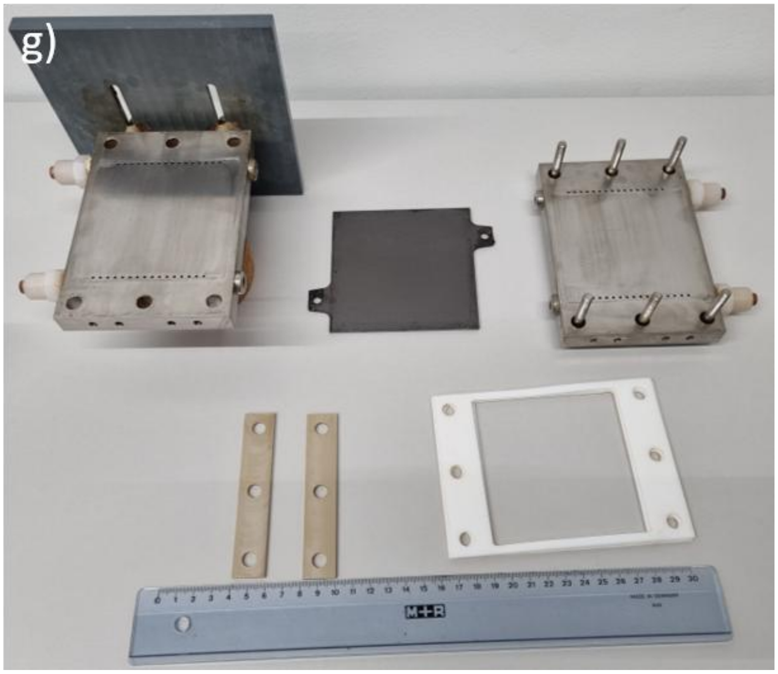

Figure S1: Summary of the construction of the cell. Scale is given in cm. a) Stainless-steel cathode with connecting screws. b) Cathode with Teflon spacers. c) Cathode with Teflon spacers and peek bars. d) Cell with closed first electrolysis compartment after installation of the glassy carbon anode. e) Installation of second set of Teflon spacers on top of anode. f) fully assembled cell. g) Individual components of the employed cell.

2.2 Custom-built Cell

The custom-built reactor also featured a sandwich geometry with a central glassy carbon plate (Sigradur® G, HTW Hochtemperatur-Werkstoffe GmbH). Figure S2 shows parts of the cell and the assembly.

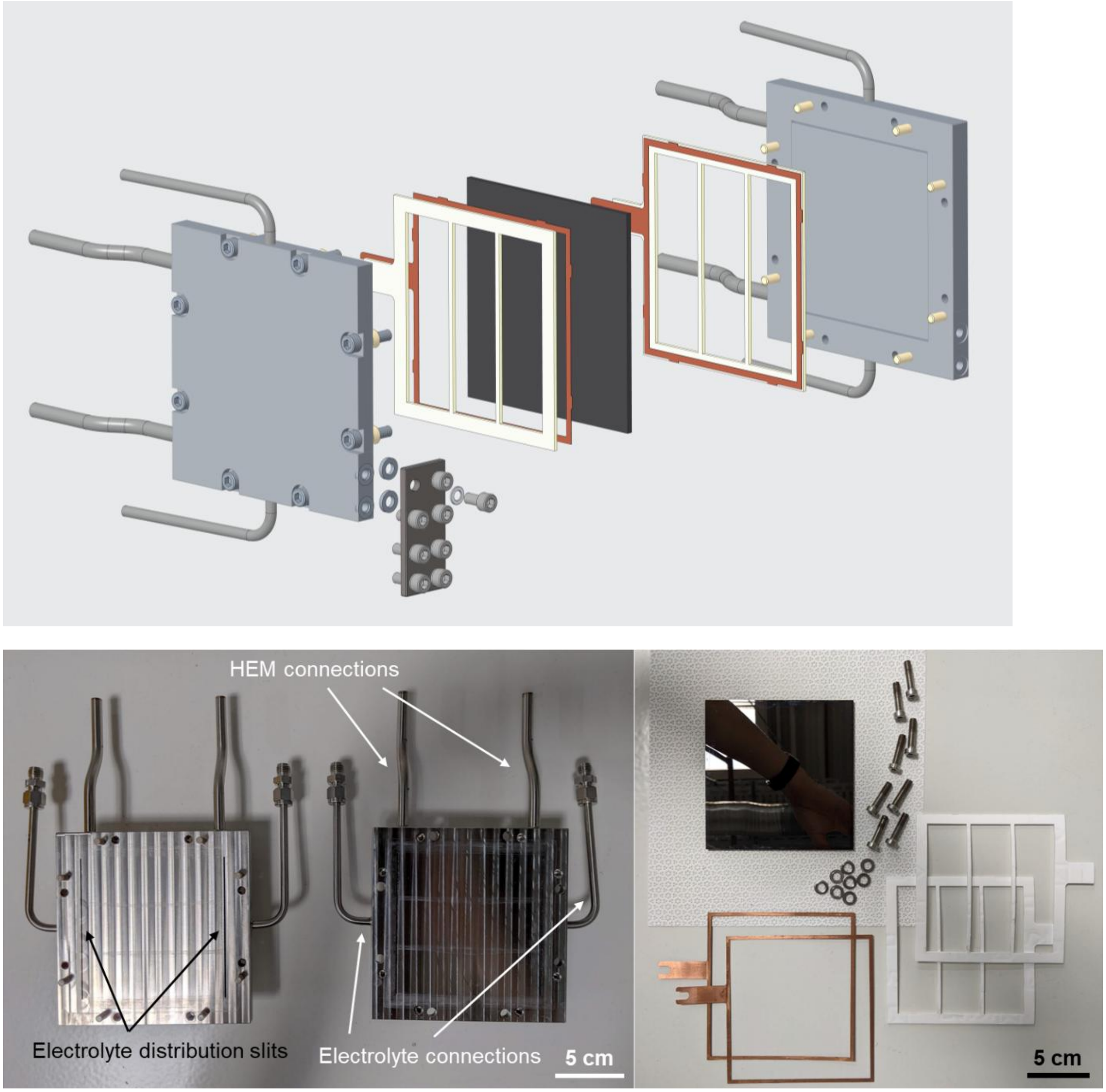

Figure S2. Custom-built reactor design. Top: Exploded view of the reactor. Bottom left: Stainless-steel cathode plates with integrated heat exchanger and fluid connections (realized by selective laser melting followed by final machining at Fraunhofer IMM). PEEK guiding pins are inserted in the left plate. Bottom right: Glassy carbon anode plate, copper contact frames, EPTFE gaskets, screws and washers for assembly.

3. Optimization of Reaction Conditions

3.1 General Reaction Protocol for Optimization

In a 250 mL Schott flask, 20 mmol of amide and sodium bromide (40-100 mmol) were weighed in. Subsequently, 40 mL of methanol and 160 mL of acetonitrile were added and the mixture was heated to the reaction temperature while stirring. Subsequently, the reaction mixture was pumped through one part of the sandwich cell. Once the entire electrolysis loop was filled, the electrolysis was started. The hydrogen evolved during the reaction was released into the ambient air.
At the end of the electrolysis, the entire solution was returned to the Schott flask and post-stirred for 2h at room temperature. Subsequently, a 10 mL aliquot was taken and the solvent evaporated from it. Subsequently mesitylene (1 mmol) and $CDCl_3$ (1 mL) were added and the yield quantified by NMR.

3.2 Initial Reaction

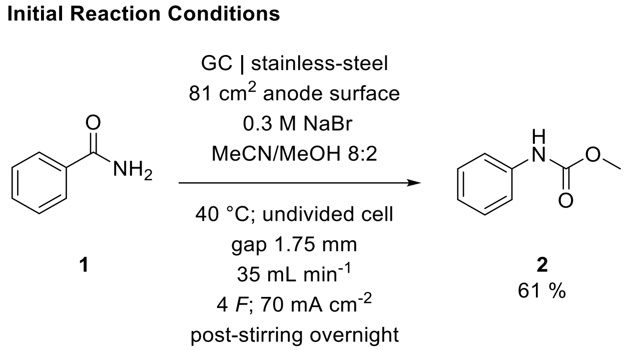

Scheme S1: Initial electrolysis conditions for the synthesis of methyl *N*-phenylcarbamate

For an initial assessment, the reaction conditions from literature[2] were adapted to the larger plate-and-frame reactor. The flowrate was adjusted to result in an identical residence time of 0.42 s.

3.3 Influence of Post-stirring Time on Yield

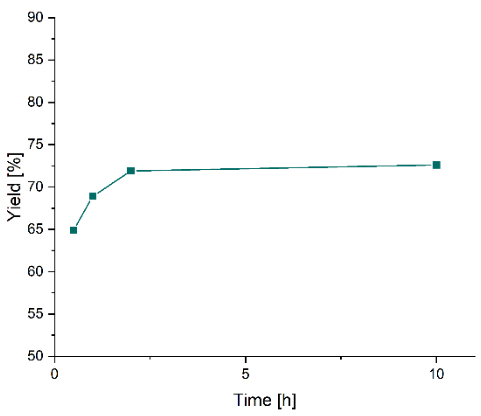

We also investigated the influence of the post-stirring time on the reaction yield. For this, a reaction was performed as described in section 3.1, with the exception that aliquots were taken from the reaction mixture after 30 min, 1h, 2h and after stirring overnight. The results of this investigation showed that the yield of carbamate still increased within the first two hours, at which point it reached a maximum. As such, a post-stirring time of 2 h was used for all following experiments.

Figure 3: Evolution of the NMR yield of 2 with different post stirring times.

3.4 Method A

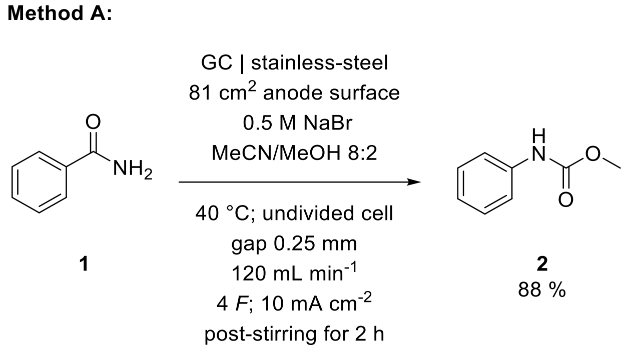

Scheme S2: Electrolysis conditions for optimized yield of methyl *N*-phenylcarbamate

The yield-oriented optimization of the rearrangement of benzamide was performed using a design of experiment approach with a 2-level factorial design generated using the Design-Expert Software by Stat-Ease. The optimization was performed with a focus on current density, NaBr concentration, interelectrode gap and flowrate. All experiments were performed in duplicate to ensure reproducibility.

The performed experiments and corresponding yields are given in table S1 and the resulting main effect plot can be seen in Figure S2. The final, optimum conditions are depicted in Scheme S2

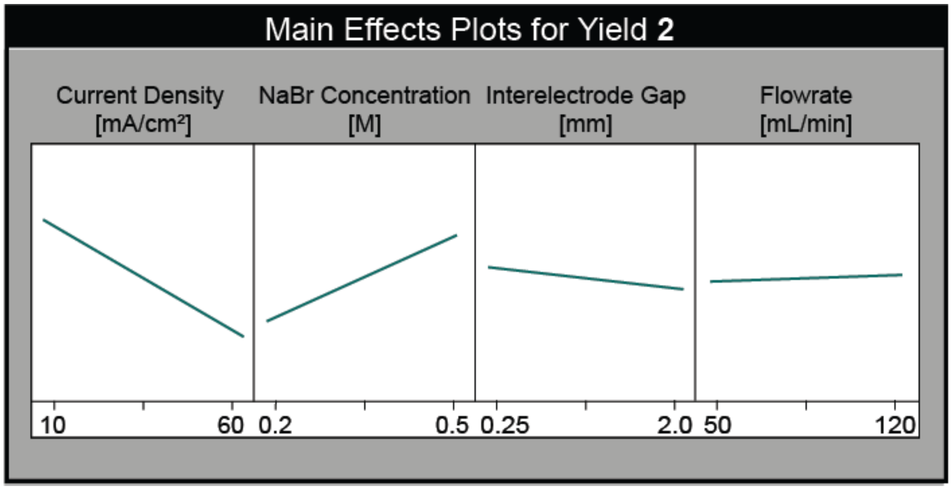

Figure S4: Main effect plots for the yield optimization screening for the formation of 2

Table S1: Screening experiments performed during the yield optimization of 2

Current density [mA/cm²]	NaBr concentration [M]	Interelectrode gap [mm]	Flowrate [mL/min]	Yield 1 [%]	Yield 2 [%]
10	0.2	0.25	120	25	19
10	0.5	0.25	50	84	78
50	0.5	2.0	120	45	49

50	0.2	2.0	120	49	47
10	0.2	2.0	120	45	47
10	0.2	0.25	120	51	52
10	0.5	2.0	120	81	84
50	0.5	0.25	120	45	55
10	0.5	0.25	120	74	72
50	0.2	2.0	50	40	45
10	0.5	2.0	50	59	54
50	0.5	0.25	50	52	52
50	0.5	2.0	50	31	29
50	0.2	0.25	120	66	59
10	0.2	2.0	50	52	52
50	0.2	0.25	50	35	37
30	0.35	1.25	85	55	62

3.5 Method B

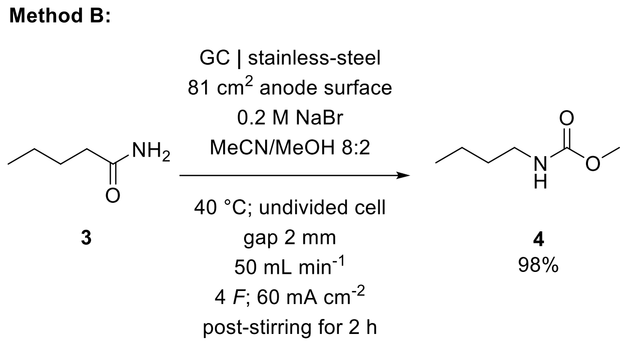

Scheme S3: Electrolysis conditions for optimized yield of methyl *N*-butylcarbamate

The yield-oriented optimization of the rearrangement of benzamide was performed using a design of experiment approach with a 2-level factorial design generated using the Design-Expert Software by Stat-Ease. The optimization was performed with a focus on current density, NaBr concentration and interelectrode gap, since flowrate had been found to have little influence in the optimization of method A. All experiments were performed in duplicate to ensure reproducibility. The performed experiments and corresponding yields are given in table S2 and the resulting main effect plot can be seen in Figure S3. The final, optimum conditions are displayed in Scheme S3

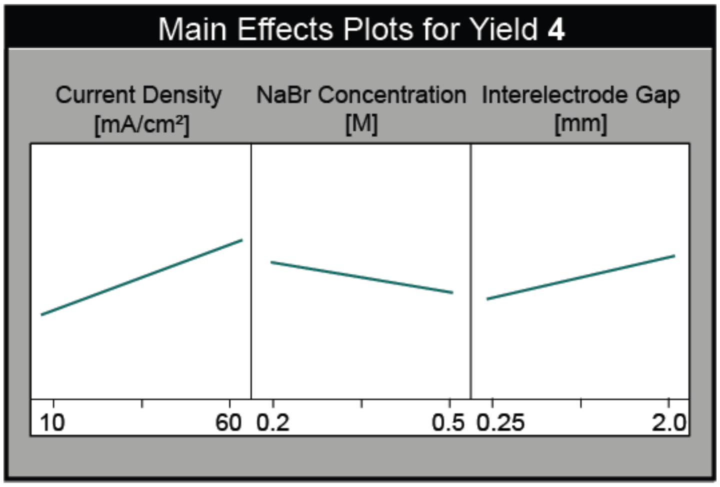

Figure S5: Main effect plots for the yield optimization screening for the formation of 4

Table S2: Screening experiments performed during the yield optimization of 4

Current density [mA/cm^2]	NaBr concentration [M]	Interelectrode gap [mm]	Yield 1 [%]	Yield 2 [%]
50	0.5	2.0	82	85
10	0.2	2.0	82	80
50	0.5	0.25	77	74
10	0.5	0.25	56	58
10	0.5	0.25	79	88
10	0.2	2.0	71	79
50	0.2	0.25	84	77
50	0.2	2.0	99	96
30	0.35	1.25	94	96

3.6 Method C

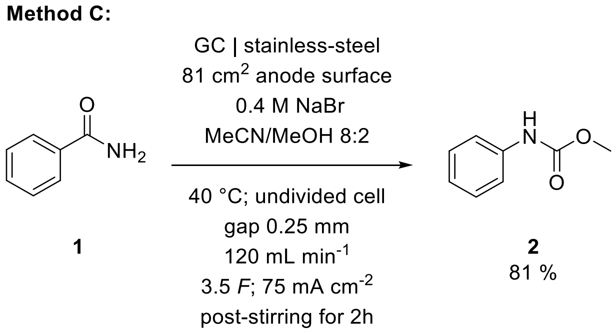

Scheme S4: Electrolysis conditions for optimized productivity of methyl *N*-phenylcarbamate

The productivity-oriented optimization of the rearrangement of benzamide was performed using a design of experiment approach with a 2-level factorial design generated using the Design-Expert Software by Stat-Ease. The optimization of the productivity of the synthesis of methyl *N*-phenylcarbamate **2** was performed with a focus on current density, starting material concentration, interelectrode gap and flow

rate. All experiments were performed in duplicate to ensure reproducibility. The performed experiments and corresponding yields and productivities are given in table S3 and the resulting main effect plot can be seen in Figure S4

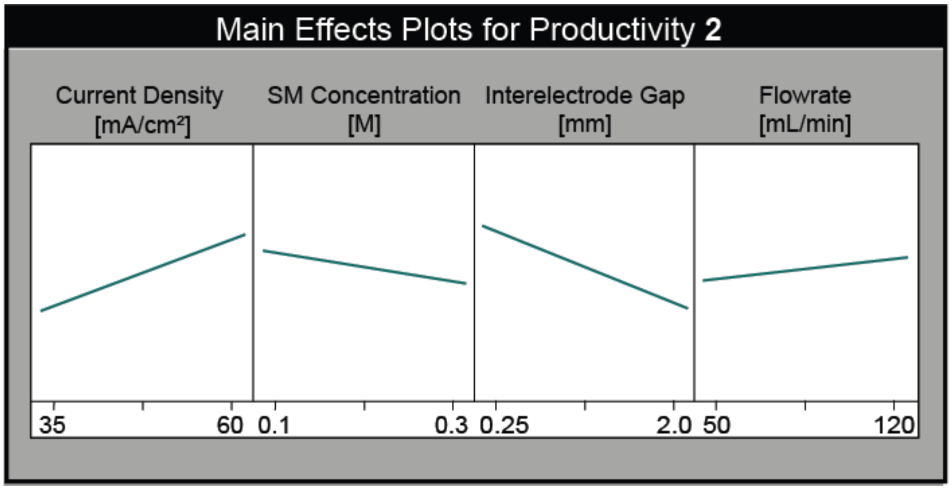

Figure S6: Main effect plots for the yield optimization screening for the formation of 4

Table S3: Screening experiments performed during the yield optimization of 2

Current density [mA/cm^2]	Concentration 1 [M]	Interelectrode gap [mm]	Flowrate [mL/min]	Yield [%]	Productivity [mmol/h]
35	0.1	0.25	50	73<br>82	20.4<br>22.9
75	0.1	0.25	120	78<br>75	47.9<br>48.0
35	0.3	0.25	120	74<br>88	20.7<br>24.6
75	0.3	0.25	50	80<br>82	38.8<br>39.8
35	0.1	2.25	120	52<br>41	14.55<br>11.47
75	0.1	2.25	50	47<br>46	23.4<br>22.9
35	0.3	2.25	50	16<br>24	4.5<br>6.7
75	0.3	2.25	120	26<br>40	14.6<br>22.4
55	0.2	1.25	85	40<br>39	16.8<br>16.4

3.7 Method D

Conditions from a previous publication[2] served as the starting point for the experiments in the custom reactors. In a small prototype version of the parallel plate reactor described previously[3] using a glassy carbon anode, the influence of temperature, flow rate and current density on the product yield and composition was investigated (Table S4). A temperature of 50 °C at 100 mA/cm² gave the best results in the preliminary tests.

Table S4: Preliminary tests for the custom made parallel plate reactor. Each electrolysis was run to a charge of 3 *F*. Electrolyte: 0.3 M NaBr in MeCN/MeOH (7:3). Substrate: 0.1 M Benzamide.

Current density [mA/cm²]	Flow rate [L/min]	Temperature [°C]	Yield [%]		
			Benzamide	Methylbenzoate	Methyl-*N*-phenylcarbamate
100	2	RT	2.6	26.9	68.1
100	2	40	7.2	7.5	84.1
100	2	50	1.4	2.7	89.1
150	2	40	3.8	6.0	79.9
100	4	40	1.0	15.8	74.2

When the full scaled cell described above was manufactured these conditions were adopted to the available setup by reducing the flow rate to 1.2 L/min, leaving temperature and current density unchanged.

The electrochemical reaction was scaled up to 500 mmol to further access method robustness. Extending the reaction time to 10 F resulted in a significant decrease in the yield of Methyl-N-phenylcarbamate. Reducing the current density to 80 mA/cm^2 improved the yield to 94% and reduced the formation of by-product to 3%. Varying the flow rate had little effect on the reaction result. When the reaction scale was further increased to 1200 mmol, a slower reaction rate was observed. After 7 F, 11% of Benzamide remained unreacted, although no brominated by-product was detected.

Table S5: Further scaling up tests for the custom made parallel plate reactor. Electrolyte: 0.35 M NaBr in MeCN/MeOH (7:3). Substrate: 500 mmol, 0.1 M Benzamide.

Current density [mA/cm²]	Flow rate [L/min]	Applied Charge [*F*]	Yield [%]		
			Benzamide	Methyl-*N*-phenylcarbamate	Methyl (4-bromophenyl)carbamate
100	1.2	4	0	85	12
100	1.2	10	0	68	22
80	1.2	3.5	0	94	3
80	2.4	4.5	0	93	4
	(1200 mmol)				
80	1.2	7	11	82	0

3.8 Method E

The best conditions used for the **1** (100 mA/cm², 50 °C, 2 L/min) substrate were adopted and gave satisfying results, making further optimization not necessary. A typical electrolysis protocol is given here:

The electrolyte solution was prepared by dissolving NaBr in MeOH and adding MeCN to the solution. The starting material (100 mmol) and the electrolyte (1 L) were added to a heated reservoir and recirculated through the system using a small centrifugal pump until the temperature was above 45 °C. Then the electrolysis was started, and samples were taken at t_0 and regular intervals until the calculated amount of current had flown. The electrolysis was stopped, and the reaction mixture transferred to a flask and stored until work up. The samples were analyzed by gas chromatography.

The reaction mixture was evaporated to one third of its volume under reduced pressure and the precipitated NaBr was filtered of under suction. The solid was washed with small volumes of MeCN and the solvents were evaporated under reduced pressure. In the case of valeramide the crude product contained no major impurities (GC-FID purity: 97.3%, Yield 97.8%) and no further purification was performed.

4. Synthesis of Carbamates at Hectogram Scale

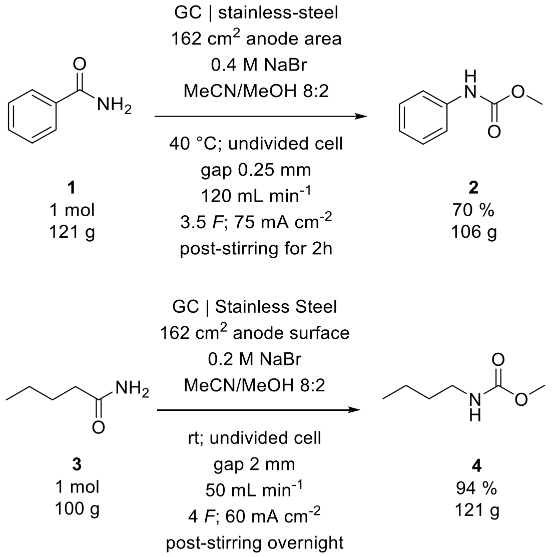

Scheme S5: Summary of the graphical summary of the methods used in the hectogram scale synthesis of carbamate

4.1 Hectogram Synthesis of Methyl *N*-phenylcarbamate

Two 5L Schott bottles were used as reaction reservoirs. To each bottle, benzamide (0.5 mol, 60.5 g) and NaBr (2mol, 206 g) were added. Subsequently, methanol (1 L) and acetonitrile (4 L) were added and the mixture was heated to 40 °C while stirring. Once the reservoirs had reached their desired temperature, the pumping circuits were filled, followed by the start of the electrolysis. The cell temperature was recorded in 1 h intervals and at the same time, 1 mL of solution was taken for analysis by NMR. Once the

full 3.5 *F* had passed, the solution was left to stir overnight. Subsequently, the solvent was removed and the residual yellow solid was extracted with ethylacetate. The resulting solution was then washed with brine and dried over $MgSO_4$.

This solution was then upconcentrated and the product crystallized by addition of cyclohexane. This yielded the product as colorless needles (106 g, 0.70 mol, 70%).

The residual solid after extraction with ethylacetate was recrystallized from water and dried at 60°C overnight in a vacuum oven, resulting in 98% (3.92 mol, 404 g) of recovered sodium bromide. Figure S7 shows the full current and voltage development over the course of the electrolysis.

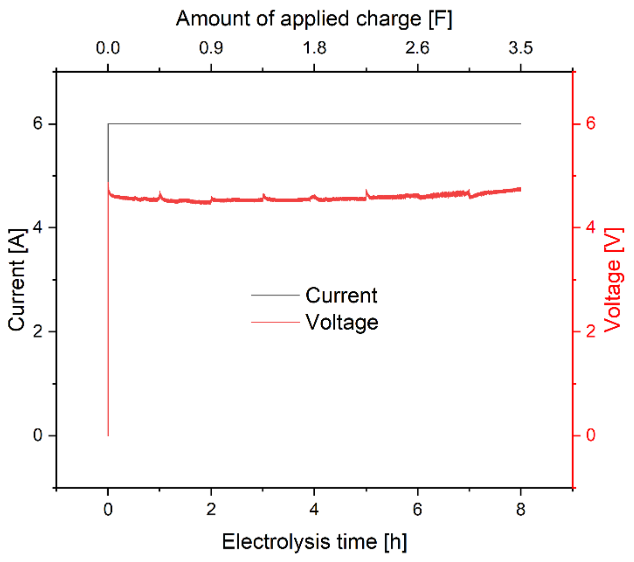

Figure S7: Current and voltage plots for the 8 h electrolysis for the hectogram synthesis of 2

4.2 Hectogram Synthesis of Methyl *N*-butylcarbamate

Two 5L Schott bottles were used as reaction reservoirs. To each bottle, pentanamide (0.5 mol, 50 g) and NaBr (1 mol, 103 g) were added. Subsequently, methanol (1 L) and acetonitrile (4 L) were added and the mixture was stirred until all solids had dissolved, the pumping circuits were filled, followed by the start of the electrolysis. The cell temperature was recorded in 1 h intervals and at the same time, 1 mL of solution was taken for analysis by NMR. Once the full 4 *F* had passed, the solution was left to stir overnight. Subsequently, the solvent was removed and the slurry was extracted with ethylacetate. The ethylacetate was subsequently removed and the product distilled off according to literature values.[4] This yielded the product as a colorless liquid (121 g, 0.94 mol, 94%).

The residual solid after extraction with ethylacetate was recrystallized from water and dried at 60°C overnight in a vacuum oven, resulting in 96% (1.92 mol, 198 g) of recovered sodium bromide. Figure S8 shows the full current and voltage development over the course of the electrolysis.

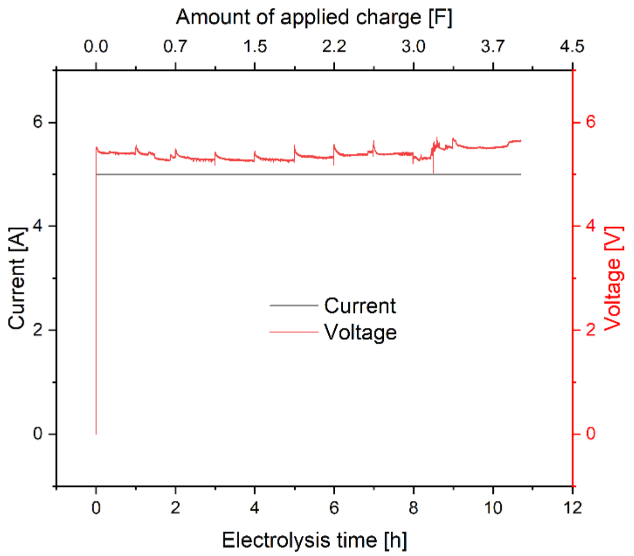

Figure S8: Current and voltage plots for the 8 h electrolysis for the hectogram synthesis of 2

5. Full Comparison of Different Flow Reactors used for the Electrochemical Hofmann Rearrangement

Cell type	IKA Flow Cell[2]		Condias SSZ 100		Custom-built Cell	
Cell Dimensions [LxWxH, cm]	4x4x10		10x3.7x14		14x2.9x14	
Active Anode Area [cm2]	12		162		200	
Rearrangement of	**1**	**3**	**1**	**3**	**1**	**3**
Flow Rate [mL/min]	5	5	120	50	1200	2000
Productivity [mmol/h]	9	4	105	89	160	182
Electricity Consumption [Wh/mol]	337.7	643.2	440.9	578.9	431.5	568.2
Electricity Consumption [kWh/kg]	2.24	4.91	2.92	4.42	2.86	4.34

6. NMR Spectra of Products

Methyl *N*-phenylcarbamate 2: ^{1}H NMR (400 MHz, $CDCl_3$): δ 7.38 (d, 2H), 7.27 (t, 2H), 7.04 (t, 1H), 6.99 (br, 1H), 3.76 (s, 3H); ^{13}C NMR (101 MHz, $CDCl_3$): δ 154.52, 138.06, 128.95, 123.39, 118.93, 52.25; The spectroscopic data match those previously reported in literature.[5]

Methyl *N*-butylcarbamate 4: ^{1}H NMR (400 MHz, $CDCl_3$): δ 4.93 (br, 1H), 3.66 (s, 3H), 3.16 (t, 2H), 1.46 (m, 3H), 1.34 (m, 2H), 0.92 (t, 3H); ^{13}C NMR (101 MHz, $CDCl_3$): δ 157.29, 51.92, 40.83, 32.14, 19.94, 13.76; The spectroscopic data match those previously reported in literature.[5]

Methyl *N*-(4-bromophenyl)carbamate: Synthesized according to literature procedure.[2] ^{1}H NMR (400 MHz, $CDCl_3$): 7.43 – 7.38 (m, 2H), 7.31 – 7.24 (m, 2H), 6.63 (s, 1H), 3.77 (s, 3H); ^{13}C NMR (400 MHz, $CDCl_3$): δ 153.94, 137.09, 132.14, 120.34, 116.12, 52.64; The spectroscopic data match those previously reported in literature.[5]

Methyl *N*-phenylcarbamate 2: ^{1}H NMR (400 MHz, $CDCl_3$)

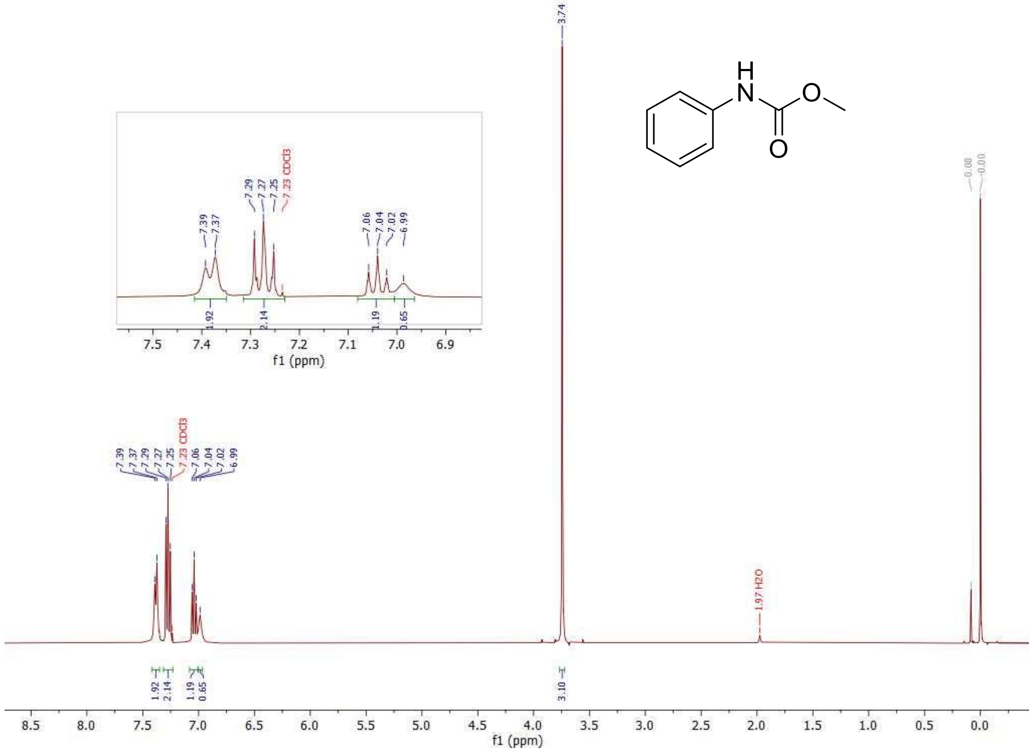

^{13}C NMR (101 MHz, $CDCl_3$):

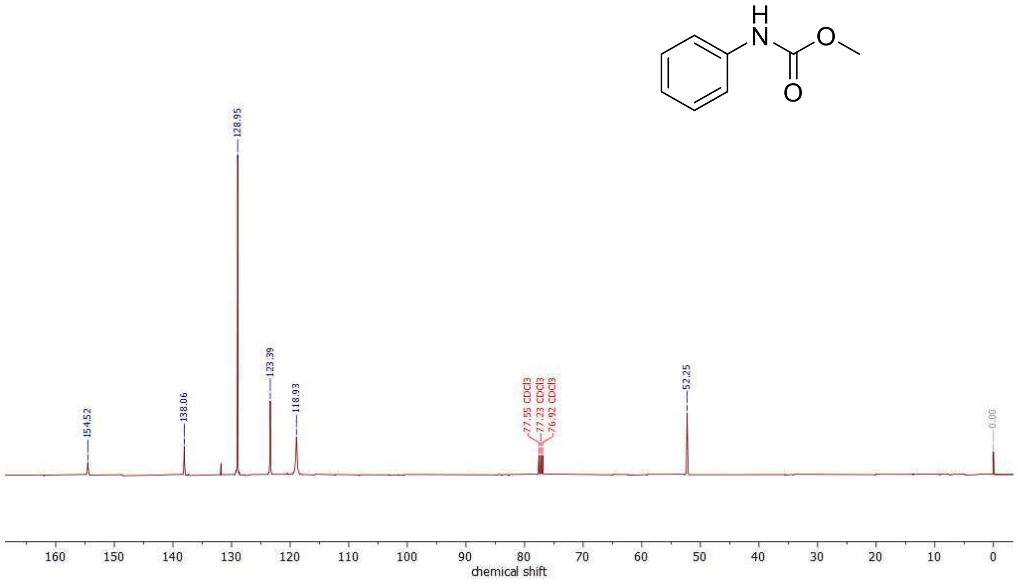

Methyl *N*-butylcarbamate 4: ^{1}H NMR (400 MHz, $CDCl_3$)

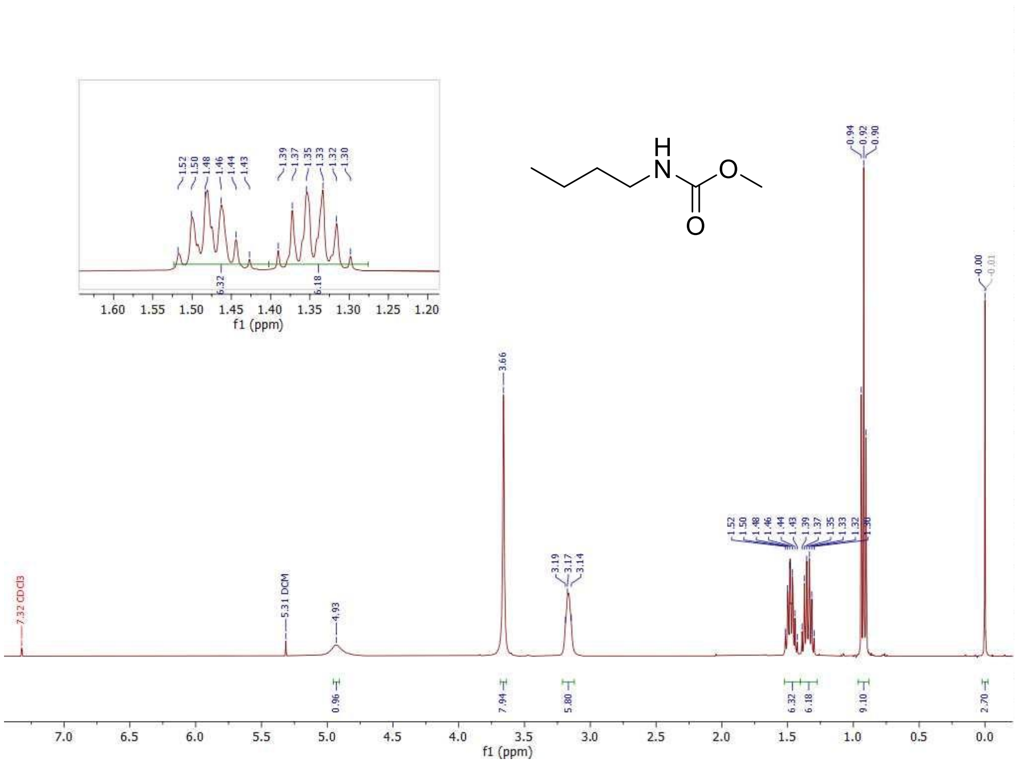

^{13}C NMR (101 MHz, $CDCl_3$):

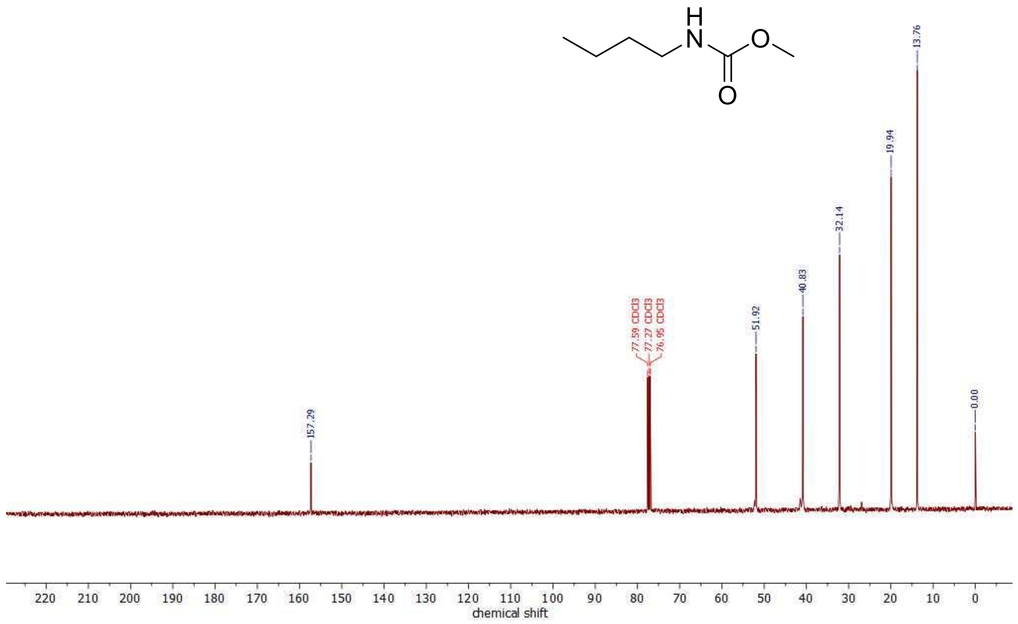

Methyl ***N*-** **(4-bromophenyl)carbamate**: ^{1}H NMR (400 MHz, $CDCl_3$)

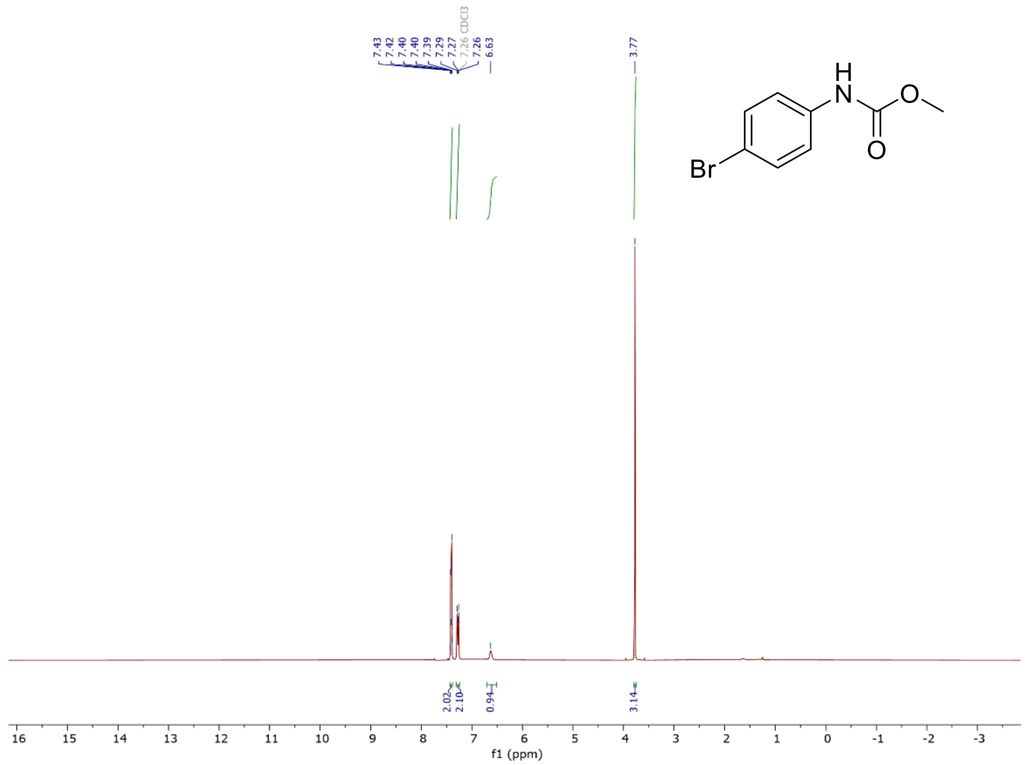

^{13}C NMR (101 MHz, $CDCl_3$):

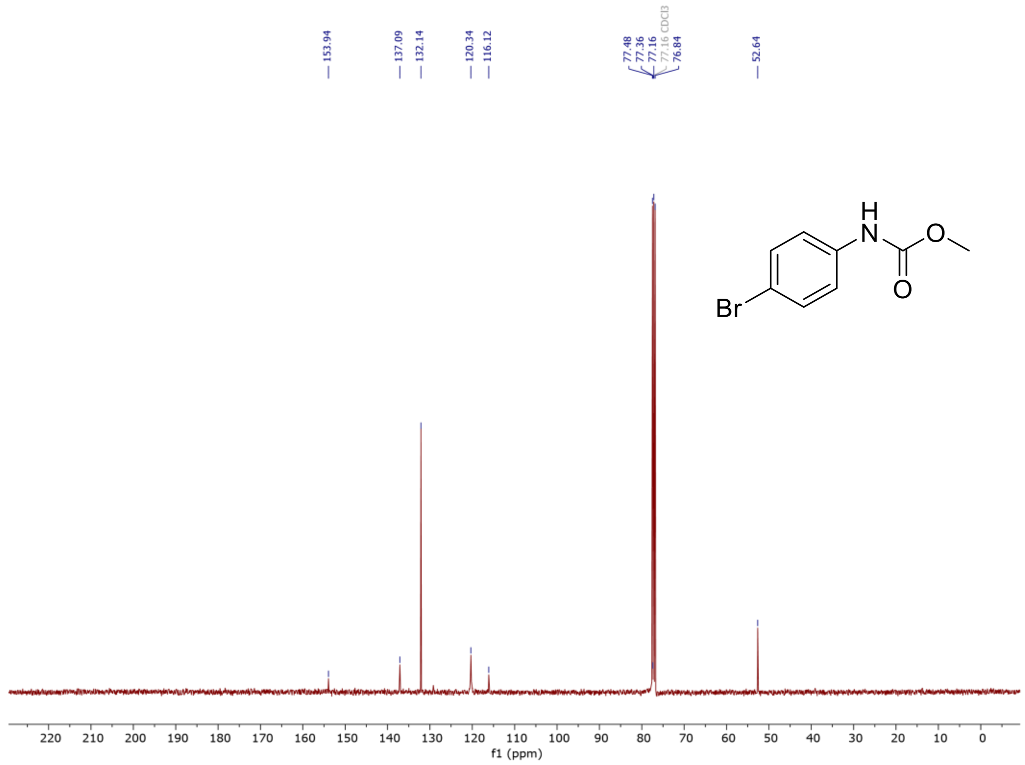

7. References

(1) https://www.condias.de/en-gb/products/SSZ_series. (accessed 09.05.2025 2025).
(2) Nater, D. F.; Hendriks, P.; Waldvogel, S. R. Electrochemical Hofmann rearrangement at high current densities in a simple flow setup. *Molecular Catalysis* **2024**, *554*, 113823. DOI: 10.1016/j.mcat.2024.113823.
(3) Ziogas, A.; Belda, J.; Kost, H.-J.; Magomajew, J.; Sperling, R. A.; Wernig, P. Peroxodicarbonate: Electrosynthesis and first directions to green industrial applications. *Current Research in Green and Sustainable Chemistry* **2022**, *5*. DOI: 10.1016/j.crgsc.2022.100341.
(4) Fochi, R.; Degani, I.; Magistris, C. An Easy and Efficient One-Step Procedure for the Preparation of Alkyl and Aryl Alkylcarbamates fromS-MethylN-Alkylthiocarbamates. *Synthesis* **2008**, *2008* (18), 2919-2924. DOI: 10.1055/s-2008-1067233.
(5) Li, L.; Xue, M.; Yan, X.; Liu, W.; Xu, K.; Zhang, S. Electrochemical Hofmann rearrangement mediated by NaBr: practical access to bioactive carbamates. *Org Biomol Chem* **2018**, *16* (25), 4615-4618. DOI: 10.1039/c8ob01059e.
